# Supplementary material for: Content-rich biological network constructed by mining PubMed abstracts
Source: BMC Bioinformatics. 2004 Oct 8;5:147. doi: 10.1186/1471-2105-5-147 (PMC528731; doi:10.1186/1471-2105-5-147)
Supplement: Additional File 2 — The original results of the above study (non-essential files are deleted to keep the file size under the limit set by BMC bioinformatics). [file 1471-2105-5-147-S2.bz2 › chilibotAdditionalFile2/dip30/393ID8700872E9879/html/FLT4_VEGFC.html]

 


 **FLT4** and **VEGFC** 
  
Found 127 abstracts in PubMed, retrieved 30.  
 

 What does Google say? 
 PDF only 
| .edu only 

---

**Interactive relationship** (e.g. stimulation, inhibition, etc)

**Stimulatory relationship**- VEGF C  [ **VEGFC** ]  promotes intratumoral lymphangiogenesis via VEGFR 3  [ **FLT4** ] , resulting facilitated invasion of cancer cells into the lymphatic vessels.  Ref: 12839674 Zhonghua Bing Li Xue Za Zhi, 2003
- Interestingly, VEGF C  [ **VEGFC** ]  stimulation of LECs induced also the formation of VEGFR 3  [ **FLT4** ]  VEGFR 2 heterodimers, in which VEGFR 3  [ **FLT4** ]  was phosphorylated only at three of the five sites while the two most C terminal tyrosine residues appeared not to be accessible for the VEGFR 2 kinase.  Ref: 12881528 J Biol ChemJ Biol Chem, 2003
- Their CM VEGF C  [ **VEGFC** ]  and VEGF D increase in response to plasma cell CM and trigger plasma cell proliferation via VEGFR 3  [ **FLT4** ] .  Ref: 12604407 Haematologica, 2003
**Neutral relationship**- A strong correlation was found between VEGF C  [ **VEGFC** ]  and VEGF D detected in carcinoma and VEGFR 3  [ **FLT4** ]  detected in neighbouring endothelial cells.  Ref: 12610509 Br J Cancer, 2003
- VEGFR 3  [ **FLT4** ]  DC in normal corneas are VEGF C  [ **VEGFC** ]  neuropilin 2, but express VEGF C  [ **VEGFC** ]  in inflammation.  Ref: 12819011 Am J Pathol, 2003
- Full length VEGF C  [ **VEGFC** ]  and VEGF D induced predominantly and the selective VEGFR 3  [ **FLT4** ]  ligand VEGF C  [ **VEGFC** ]  156S exclusively lymphangiogenesis.  Ref: 12714562 Circ Res< MedlineTA>Circ Res, 2003
- A functional VEGF C  [ **VEGFC** ]  autocrine growth loop was demonstrated in mesothelioma cells by targeting VEGF C  [ **VEGFC** ]  expression and binding to VEGFR 3  [ **FLT4** ] .  Ref: 12594815 Int J Cancer, 2003
- Secondly, ectopic expression in the weakly metastatic NM 081 cell line of a mutant form of VEGF C  [ **VEGFC** ]  that is only able to activate VEGFR 3  [ **FLT4** ]  strongly promoted metastasis of these cells to the regional lymph nodes and lung.  Ref: 12566318 Cancer Res, 2003
- Genetic analysis of families with Milroy s disease identified mutations in VEGFR3  [ **FLT4** ]  as a cause of congenital lymphedema, confirming the importance of  **VEGFC**  VEGFR3  [ **FLT4** ]  signaling in lymphatic development.  Ref: 12543715 Ann N Y Acad Sci, 2002
- BACKGROUND Vascular endothelial growth factor C  [ **VEGFC** ]  VEGF C  [ **VEGFC** ]  plays an important role in lymphangiogenesis and activates VEGF receptor 3 VEGFR 3  [ **FLT4** ] .  Ref: 12518370 Cancer, 2003
- They belong to the larger family which also includes VEGF, placenta growth factor PlGF and VEGF B, VEGF C  [ **VEGFC** ]  and VEGF D are ligands for the endothelial cell specific tyrosine kinase receptors VEGFR 2 and VEGFR 3  [ **FLT4** ] .  Ref: 12888864 Thromb Haemost, 2003
- Two growth factors, vascular endothelial growth factor C  [ **VEGFC** ]  VEGF C  [ **VEGFC** ]  and D VEGF D, have been found to be important in the proper formation and maintenance of the lymphatic network, through their receptor VEGFR 3  [ **FLT4** ] .  Ref: 12543720 Ann N Y Acad Sci, 2002
- Firstly, ectopic expression of a soluble VEGFR 3  [ **FLT4** ]  receptor globulin protein in MT 450 tumor cells that are highly metastatic via the lymphatics blocked VEGF C  [ **VEGFC** ]  and VEGF D activity and suppressed metastasis formation in both the regional lymph nodes and the lungs.  Ref: 12566318 Cancer Res, 2003

**Non-interactive relationship** (e.g. studied together, co-existance, homology, etc.)

- Vessels in the parental tumours were found to express VEGFR 3  [ **FLT4** ] , the receptor for VEGF C  [ **VEGFC** ]  and VEGF D.  Ref: 12778077 Br J Cancer, 2003
- OBJECTIVE To investigate the relationship between vascular endothelial growth factor C  [ **VEGFC** ]  VEGF C  [ **VEGFC** ]  expression, VEGFR 3  [ **FLT4** ]  expression, lymphangiogenesis and angiogenesis in human non small cell lung cancer NSCLC.  Ref: 12875690 Chin Med J (Engl), 2003
- We have studied the expression of VEGFR 3  [ **FLT4** ] , its ligand VEGF C  [ **VEGFC** ]  and the co receptor neuropilin 2, in normal and inflamed corneas and characterized the phenotype and distribution of VEGFR 3  [ **FLT4** ]  cells.  Ref: 12819011 Am J Pathol, 2003
- Clinical significance of co expression of VEGF C  [ **VEGFC** ]  and VEGFR 3  [ **FLT4** ]  in non small cell lung cancer.  Ref: 12875690 Chin Med J (Engl), 2003
- The aim of this work was to determine 1 the distribution of VEGF C  [ **VEGFC** ]  and VEGFR 3  [ **FLT4** ]  in CRC, and 2 the biological significance of such expression.  Ref: 12168824 Anticancer Res< MedlineTA>Anticancer Res, 2003
- Expression of the vascular endothelial growth factor receptor 3  [ **FLT4** ]  VEGFR 3  [ **FLT4** ]  and its ligand VEGF C  [ **VEGFC** ]  in human colorectal adenocarcinoma.  Ref: 12168824 Anticancer Res< MedlineTA>Anticancer Res, 2003
- CONCLUSION Real time quantitative reverse transcriptase PCR is a sensitive method for detection and quantification of VEGF C  [ **VEGFC** ]  and VEGFR 3  [ **FLT4** ]  mRNA expression.  Ref: 12660643 Surgery, 2003
- We studied the therapeutic potential of human VEGF family members VEGF A, VEGF B, VEGF C  [ **VEGFC** ] , and VEGF D as well as a VEGFR 3  [ **FLT4** ]  specific mutant VEGF C  [ **VEGFC** ]  156S using adenoviral gene transfer in rabbit hindlimb skeletal muscle.  Ref: 12714562 Circ Res< MedlineTA>Circ Res, 2003
- Modulation of VEGFR 2 mediated endothelial cell activity by VEGF C  [ **VEGFC** ]  VEGFR 3  [ **FLT4** ] .  Ref: 12393458 Blood, 2003
- Expression of VEGF C  [ **VEGFC** ]  and VEGFR 3  [ **FLT4** ]  was significantly associated with lymph node metastasis P = 0.008 and P = 0.013 respectively and lymphatic invasion P = 0.027 and P = 0.020 respectively.  Ref: 12839674 Zhonghua Bing Li Xue Za Zhi, 2003
- To avoid these blood vascular side effects of VEGF C  [ **VEGFC** ] , we constructed a viral vector for a VEGFR 3  [ **FLT4** ]  specific mutant form of VEGF C  [ **VEGFC** ]  VEGF C156S for lymphedema gene therapy.  Ref: 12235206 J Exp Med, 2002
- Studies in animal models carried out over the past three years have shown that the soluble protein growth factors, vascular endothelial growth factor VEGF C  [ **VEGFC** ]  and VEGF D, and their cognate receptor tyrosine kinase, VEGF receptor 3 VEGFR 3  [ **FLT4** ] , are critical regulators of lymphangiogenesis.  Ref: 12386934 Bioessays, 2002
- A significant positive correlation was found between VEGF C  [ **VEGFC** ]  expression and VEGFR 3  [ **FLT4** ]  expression in cancer cells P <.  Ref: 12875690 Chin Med J (Engl), 2003
- We assessed the presence of vascular endothelial growth factor VEGF C  [ **VEGFC** ] , VEGF D and their receptor VEGFR 3  [ **FLT4** ]  by immunohistochemistry in 59 epithelial ovarian carcinomas, 11 borderline tumours and 20 benign cystadenomas.  Ref: 12610509 Br J Cancer, 2003
- BACKGROUND This study was undertaken to determine whether vascular endothelial growth factor C  [ **VEGFC** ]  VEGF C  [ **VEGFC** ]  and the expression of its receptor VEGF receptor 3 VEGFR 3  [ **FLT4** ]  are correlated with lymph node metastasis in human colorectal cancer and whether their expression levels might be used to predict lymph node metastasis.  Ref: 12660643 Surgery, 2003
- VEGF C  [ **VEGFC** ]  and its cognate receptor VEGFR 3  [ **FLT4** ]  are co expressed in mesothelioma cell lines.  Ref: 12594815 Int J Cancer, 2003
- We measured the gene expression of VEGF ligands VEGF A, VEGF B, VEGF C  [ **VEGFC** ] , and VEGF D and their receptors VEGFR 1, VEGFR 2, and VEGFR 3  [ **FLT4** ] , in normal colorectal tissues n = 20, adenomas n = 10, and in CRC n = 71 representing different Duke s stages using ribonuclease protection assay, semi quantitative relative reverse transcriptase polymerase chain reaction, together with the pattern of their expression by immunohistochemistry.  Ref: 12754739 J Pathol< MedlineTA>J Pathol, 2003
- Vascular endothelial growth factor C  [ **VEGFC** ]  and vascular endothelial growth factor receptor 3  [ **FLT4** ]  expression in squamous cell carcinomas of the head and neck.  Ref: 12784238 Head Neck, 2003
- We conclude that VEGF C  [ **VEGFC** ] , VEGF D and VEGFR 3  [ **FLT4** ]  play an important role in lymphatic spread and intraperitoneal tumour development in ovarian carcinoma.  Ref: 12610509 Br J Cancer, 2003
- The VEGFR 3  [ **FLT4** ]  specific mutant form of VEGF C  [ **VEGFC** ]  called VEGF C156S lacks blood vascular side effects but is sufficient for therapeutic lymphangiogenesis in a mouse model of lymphedema.  Ref: 12888864 Thromb Haemost, 2003
- RESULTS Of the 76 NSCLC cases, 55 were VEGF C  [ **VEGFC** ]  positive and 40 were VEGFR 3  [ **FLT4** ]  positive in cancer cells.  Ref: 12875690 Chin Med J (Engl), 2003
- Immunohistochemistry in 18 HNSCC specimens assigned strong to mediate VEGF C  [ **VEGFC** ]  IR and minor VEGFR3  [ **FLT4** ]  IR to tumor cells and strong VEGF C  [ **VEGFC** ]  and VEGFR3  [ **FLT4** ]  IR to tumor surrounding vessels.  Ref: 12784238 Head Neck, 2003
- METHODS Samples of 76 NSCLC cases with the neighboring noncancerous tissue were studied using anti VEGF C  [ **VEGFC** ] , VEGFR 3  [ **FLT4** ]  and CD34 antibodies.  Ref: 12839674 Zhonghua Bing Li Xue Za Zhi, 2003
- Vascular endothelial growth factor VEGF receptor 3 VEGFR 3  [ **FLT4** ] , a receptor for VEGF C  [ **VEGFC** ] , was shown to be essential for angiogenesis as well as for lymphangiogenesis.  Ref: 12393458 Blood, 2003
- Immunohistochemical staining for VEGF C  [ **VEGFC** ]  and VEGFR 3  [ **FLT4** ]  was performed.  Ref: 12518370 Cancer, 2003
- The objective of the current study was to determine whether the expression of VEGF C  [ **VEGFC** ]  and VEGFR 3  [ **FLT4** ]  correlates with clinicopathologic factors and prognosis in patients with primary NSCLC.  Ref: 12518370 Cancer, 2003
- The expression and levels of the VEGF C  [ **VEGFC** ]  and VEGF D cytokines, the VEGF receptors VEGFR 2 and VEGFR 3  [ **FLT4** ] , and newly described lymphatic endothelial markers, LYVE 1, Prox 1, podoplanin and 5 nucleotidase were assessed.  Ref: 12851706 Int J Oncol, 2003
- Because HNSCC preferentially metastasizes to regional lymph nodes, we investigated the expression of VEGFR3  [ **FLT4** ]  and its ligand VEGF C  [ **VEGFC** ]  in head and neck squamous cell carcinomas by semiquantitative RT PCR 4 HNSCC cells lines and 6 HNSCC specimens and by immunohistochemistry 18 HNSCC specimens.  Ref: 12784238 Head Neck, 2003
- METHODS Seventy six NSCLC samples were stained for VEGF C  [ **VEGFC** ] , VEGFR 3  [ **FLT4** ]  and CD34 with immunohistochemical methods.  Ref: 12875690 Chin Med J (Engl), 2003
- The messenger RNA mRNA expression of VEGF C  [ **VEGFC** ]  and VEGFR 3  [ **FLT4** ]  was quantified with a new method for kinetic quantitative polymerase chain reaction PCR, real time quantitative RTQ reverse transcriptase PCR.  Ref: 12660643 Surgery, 2003
- Novel Expression of Vascular Endothelial Growth Factor Receptor VEGFR 3  [ **FLT4** ]  and VEGF C  [ **VEGFC** ]  on Corneal Dendritic Cells.  Ref: 12819011 Am J Pathol, 2003
- 0.01 and combined VEGF C  [ **VEGFC** ]  and or VEGFR 3  [ **FLT4** ]  expression both positive vs. VEGF C  [ **VEGFC** ]  or VEGFR 3  [ **FLT4** ]  positive.  Ref: 12518370 Cancer, 2003
- Recently, mRNA for VEGF C  [ **VEGFC** ]  the ligand for VEGFR 3  [ **FLT4** ] , was found to be up regulated in colorectal adenocarcinoma CRC.  Ref: 12168824 Anticancer Res< MedlineTA>Anticancer Res, 2003
- As TAMs are derived from monocytes in the circulation, a search in peripheral blood for candidate precursors of VEGFR 3  [ **FLT4** ]  expressing TAMs revealed a subfraction of CD14 positive, VEGFR 3  [ **FLT4** ]  expressing monocytes, that failed to express VEGF C  [ **VEGFC** ]  and VEGF D.  Ref: 12213723 Am J Pathol, 2002
- The capacity to induce lymphangiogenesis by manipulation of the VEGF C  [ **VEGFC** ]  VEGF D VEGFR 3  [ **FLT4** ]  signaling pathway offers new opportunities to understand the function of the lymphatic system and to develop novel treatments for lymphatic disorders.  Ref: 12386934 Bioessays, 2002
- Patients who had positive staining for both VEGF C  [ **VEGFC** ]  and VEGFR 3  [ **FLT4** ]  exhibited the most unfavorable prognoses.  Ref: 12518370 Cancer, 2003
- The clinicopathologic implications of VEGF C  [ **VEGFC** ]  and VEGFR 3  [ **FLT4** ]  expression were analyzed statistically.  Ref: 12518370 Cancer, 2003
- OBJECTIVE To study the relationship between angiogenesis and lymphangiogenesis with the expression of vascular endothelial growth factor C  [ **VEGFC** ]  VEGF C  [ **VEGFC** ]  and VEGFR 3  [ **FLT4** ]  in human non small cell lung cancer NSCLC.  Ref: 12839674 Zhonghua Bing Li Xue Za Zhi, 2003
- Clinical significance of vascular endothelial growth factor C  [ **VEGFC** ]  and vascular endothelial growth factor receptor 3  [ **FLT4** ]  in patients with nonsmall cell lung carcinoma.  Ref: 12518370 Cancer, 2003
- Targeted disruption of the VEGFR 3  [ **FLT4** ]  gene in mice and our previous study using an antagonistic monoclonal antibody MoAb for VEGFR 3  [ **FLT4** ]  suggested that VEGF C  [ **VEGFC** ]  VEGFR 3  [ **FLT4** ]  signals might be involved in the maintenance of vascular integrity.  Ref: 12393458 Blood, 2003
- Increased expression of VEGF C  [ **VEGFC** ] , VEGF D and VEGFR 3  [ **FLT4** ]  was significantly associated with lymph node metastasis and peritoneal metastasis outside the pelvis.  Ref: 12610509 Br J Cancer, 2003
- VEGF C  [ **VEGFC** ]  expression and VEGFR 3  [ **FLT4** ]  expression were positively associated with lymph node metastasis and lymphatic invasion P <.  Ref: 12875690 Chin Med J (Engl), 2003
- A number of clinical and experimental studies suggest that tumor induced lymphangiogenesis driven by vascular endothelial growth factor VEGF C  [ **VEGFC** ]  and or VEGF D induced activation of VEGF receptor VEGFR 3  [ **FLT4** ]  may promote metastasis to regional lymph nodes.  Ref: 12566318 Cancer Res, 2003
- RESULTS Of 180 patients with NSCLC, 137 patients 76.1% were positive for VEGF C  [ **VEGFC** ] , and 40 patients 22.2% were positive for VEGFR 3  [ **FLT4** ] .  Ref: 12518370 Cancer, 2003
- A significant positive correlation was found between VEGF C  [ **VEGFC** ]  in cancer cells and VEGFR 3  [ **FLT4** ]  in lymphatic endothelial cells P = 0.009.  Ref: 12839674 Zhonghua Bing Li Xue Za Zhi, 2003
- CONCLUSIONS VEGF C  [ **VEGFC** ]  and VEGFR 3  [ **FLT4** ]  expression may be indicative of survival rates for patients with NSCLC.  Ref: 12518370 Cancer, 2003
- Sections of formalin fixed and paraffin embedded tissues from 56 CRC were immunohistochemically stained for VEGF C  [ **VEGFC** ]  and VEGFR 3  [ **FLT4** ] .  Ref: 12168824 Anticancer Res< MedlineTA>Anticancer Res, 2003
- OBJECTIVE To document the pattern of expression of the two angiogenic growth factors, vascular endothelial growth factor VEGF B and VEGF C  [ **VEGFC** ] , and of their receptors, VEGFR 1, VEGFR 2, and VEGFR 3  [ **FLT4** ] , in order to investigate their possible role in the regulation of angiogenesis in normal cycling human endometrium.  Ref: 12225295 Acta Obstet Gynecol Scand, 2002
- Recent work from many laboratories has demonstrated that the vascular endothelial growth factor C  [ **VEGFC** ]  VEGF D VEGFR 3  [ **FLT4** ]  signaling pathway is crucial for lymphangiogenesis,.  Ref: 12235206 J Exp Med, 2002
- A family of growth factors highly specific for endothelial cells was identified more than 10 years ago, in which the receptor of vascular endothelial growth factor C  [ **VEGFC** ]  VEGFR 3  [ **FLT4** ]  is implicated in the regulation of lymphatic development and regeneration.  Ref: 12588961 J Histochem Cytochem, 2003
- These data demonstrate, for the first time, the expression of VEGFR 3  [ **FLT4** ]  and VEGF C  [ **VEGFC** ]  on tissue DC, which implicate a novel potential relationship between lymphangiogenesis and leukocyte trafficking in the eye.  Ref: 12819011 Am J Pathol, 2003
- Examination of the VEGF C  [ **VEGFC** ]  specific receptors VEGFR 2 KDR Flk 1 and VEGFR 3  [ **FLT4** ]  Flt 4  [ **FLT4** ]  demonstrated intense endothelial immunoreactivity for VEGFR 2, as well as VEGFR 2 and 3 expression on the majority of neoplastic cells, suggesting a possible role in autocrine paracrine neoplastic growth regulation.  Ref: 12861060 Mod Pathol, 2003
- The broad expression of VEGF C  [ **VEGFC** ]  and VEGFR3  [ **FLT4** ]  in HNSCC suggests involvement in tumor lymph angiogenesis and vascular angiogenesis, promoting tumor growth and propagation of cancer cells.  Ref: 12784238 Head Neck, 2003
- These TAMs also expressed the VEGF C  [ **VEGFC** ]  and VEGF D specific tyrosine kinase receptor VEGFR 3  [ **FLT4** ] .  Ref: 12213723 Am J Pathol, 2002
- CONCLUSIONS In NSCLC, VEGF C  [ **VEGFC** ]  and VEGFR 3  [ **FLT4** ]  are related to the lymphangiogenesis, angiogenesis, and occurrence and development of lung cancers.  Ref: 12875690 Chin Med J (Engl), 2003
- Similarly, antibodies to VEGF receptor VEGFR 2 and VEGF C  [ **VEGFC** ]  receptor VEGFR 3  [ **FLT4** ]  were synergistic in inhibiting mesothelioma cell growth.  Ref: 12594815 Int J Cancer, 2003
